# *Ganoderma lucidum* spore oil alleviates psychological stress-evoked tumor progression by enhancing FcγR-mediated macrophage phagocytosis

**DOI:** 10.1186/s13020-025-01168-0

**Published:** 2025-07-14

**Authors:** Dong-Dong Li, Zi-Xuan Li, Yang-Fan Zhou, Ling Jin, Wan-Li Liang, Wen-Dong Xu, Xiang Luo, Guan-Di Xiao, Qi-Jun Chen, Ting Xie, Hui Xu, Yang Liu, Hong-Fei Cai, Yun-Feng Cao, Wan-Yang Sun, Yi-Fang Li, Lei Liang, Ju-Yan Liu, Yan-Ping Wu, Rong-Rong He

**Affiliations:** 1 State Key Laboratory of Bioactive Molecules and Druggability Assessment/Guangdong Basic Research Center of Excellence for Natural Bioactive Molecules and Discovery of Innovative Drugs/Guangdong Engineering Research Center of Traditional Chinese Medicine & Disease Susceptibility/The First Affiliated Hospital/The Sixth Affiliated Hospital/Guangdong Second Provincial General Hospital/Guangdong Engineering Research Center of Traditional Chinese Medicine & Health Products/International Cooperative Laboratory of TCM Modernization and Innovative Drug Development of Chinese Ministry of Education (MOE)/Guangdong Province Key Laboratory of Pharmacodynamic Constituents of TCM and New Drugs Research/College of Pharmacy/School of Traditional Chinese Medicine, Jinan University, Guangzhou, 510632 China; 2 School of Pharmacy, Faculty of Medicine, Macau University of Science and Technology, Macau SAR, China; 3 National Engineering Research Center of Pharmaceutical Processing Technology of Traditional Chinese Medicine and Drug Innovation/Guangdong Provincial Key Laboratory of Medicinal Lipids, Guangzhou HanFang Pharmaceutical Company Limited, Guangzhou, 510240 China; 4 School of Chinese Materia Medica and Yunnan Key Laboratory of Southern Medicinal Utilization, Yunnan University of Chinese Medicine, Kunming, 650500 China; 5 NHC Key Laboratory of Reproduction Regulation, Shanghai Institute for Biomedical and Pharmaceutical Technologies, Shang Hai, 200032 China

**Keywords:** Psychological stress, Lysophosphatidylinositol, Macrophages, *Ganoderma lucidum* spore oil, Phagocytosis

## Abstract

**Background:**

*Ganoderma lucidum* (*G. lucidum*), has been documented as a medicinal herb in classical texts and officially recognized in both Eastern and Western pharmacopeias. *G. lucidum* spore oil (GLSO), a lipid substance extracted from sporoderm-broken spores, has shown potential in enhancing immune function and prolonging the survival of tumor patients. However, the mechanisms underlying GLSO’s immunomodulatory effects remain poorly unknown.

**Methods:**

The effect of psychological stress on tumor progression and macrophage phagocytosis was analyzed by an *in vivo* small animal imaging system and flow cytometry. The effect of psychological stress on phospholipid composition in mice was investigated by LC–MS/MS based lipidomic analysis. The effectiveness of GLSO in tumor-bearing mice subjected to restraint stress was observed by tumor burden and phagocytosis of macrophages. Finally, the underlying mechanism of GLSO on macrophage phagocytosis in mice subjected to psychological stress was explored by RNA-seq, and the FcγR/SYK-mediated macrophage phagocytosis pathway was confirmed by qPCR, Western blotting, and confocal laser technology.

**Results:**

Our study discovered that psychological stress-triggered tumor progression is contributed to by liposoluble components-impaired macrophage phagocytosis. Lipidomics analysis further identified lysophosphatidylinositol [LPI (18:0)] as a key factor suppressing macrophage phagocytic capacity under psychological stress. GLSO was shown to mitigate psychological stress-evoked tumor progression by enhancing macrophage-mediated phagocytosis of tumor cells *in vivo*. Mechanistically, transcriptomics analysis revealed that the LPI-mediated FcγR phagocytosis pathway is a crucial axis driving the therapeutic effect of GLSO under psychological stress.

**Conclusion:**

Our findings illustrate that psychological stress-promoted cancer progression is contributed by the critical liposoluble components LPI (18:0)-mediated FcγR phagocytosis signaling inhibition. GLSO alleviates the dampened phagocytosis of macrophages caused by stress through regulating LPI/FcγR-mediated phagocytosis-related pathways, underscoring its potential as a therapeutic intervention for stress-related tumor progression.

**Graphical Abstract:**

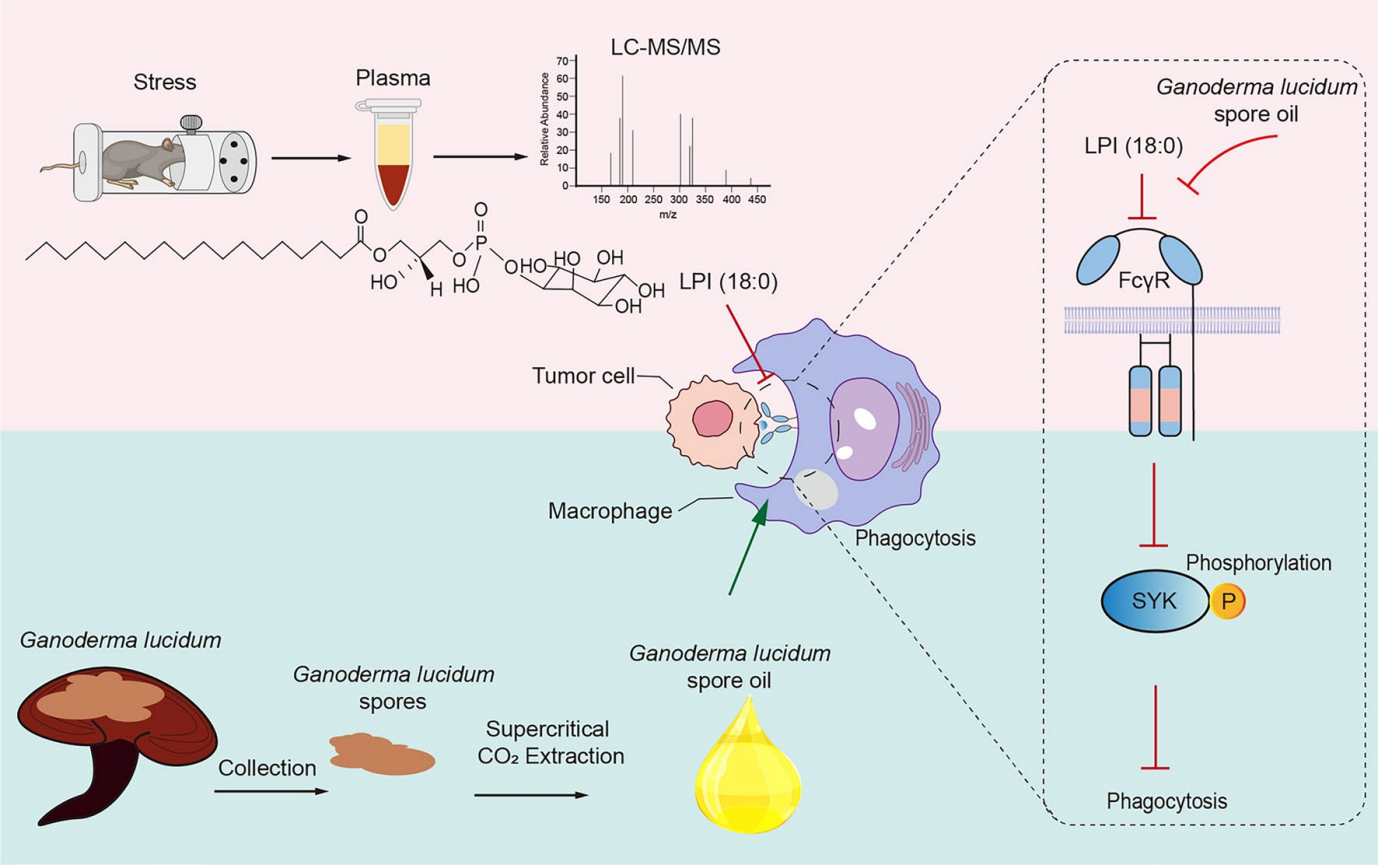

## Background

Psychological stress is often perceived as a burden [1, 2] and has been linked to suppressed immunity, increasing cancer risk [3, 4]. Accumulating clinical evidence suggests that psychological stress responses can influence cancer development, progression, and metastasis [5, 6]. Ovarian cancer (OC), one of the deadliest gynecological malignancies, continues to pose a global health challenge due to its high mortality rates [7]. Its prevalence among individuals with depression and anxiety highlights the urgent need to explore how psychological stress influences cancer progression, offering potential pathways for improved therapeutic strategies.

Recent studies have discovered that macrophages are a dominant immune cell type within the tumor microenvironment (TME), comprising 50% of cells in solid tumors and playing a critical role in tumor progression [8–12]. Macrophage phagocytosis is a complex process that involves receptor-ligand interaction, phagocytic cup formation, and closure and relies on signal transduction, cytoskeletal rearrangement, and membrane remodeling [13, 14]. Phospholipids, particularly phosphatidylinositol (PI), are pivotal in these dynamics [15, 16]. Phosphatidylcholine, a metabolite of phospholipid metabolism, is crucial for membrane integrity and lipid-dependent signaling cascades. Meanwhile, phosphorylated PI derivatives serve as signaling molecules and are directly implicated in the activation of signaling pathways associated with macrophage phagocytosis [17]. In addition, the membrane of macrophages is characterized by the presence of various phagocytic receptors, which facilitate the recognition and uptake of pathogens, cellular debris, and other exogenous materials [13, 18, 19]. These phagocytic receptors play a critical role in modulating the phagocytic activity of macrophages. Typically, Fcγ receptors enhance macrophage phagocytosis of antibody-coated pathogens through antibody binding [20, 21]. Notably, Fcγ R-mediated phagocytosis represents one of the most direct and effective mechanisms for immune clearance within the immune system [22]. Fcγ receptors mediate target attachment to macrophage membranes, initiating phagocytosis upon antigen recognition [20, 23]. However, the exact mechanism by which lipids within macrophages impact their phagocytic capabilities remains elusive.

*Ganoderma lucidum*, has been documented for millennia as a medicinal herb in Shennong Bencao Jing (The Divine Farmer’s Materia Medica) and officially recognized in both Eastern and Western pharmacopeias (Pharmacopoeia of the People’s Republic of China 2020; The United States pharmacopeia 2021), exhibits diverse pharmacological properties, especially in immunomodulatory [24–26]. Studies and some preclinical trials have indicated potential anticancer and immunoregulatory activities [27–29]. The popularity of *G. lucidum* as a complementary therapy among cancer patients has been on the rise [30]. To date, more than 300 active compounds have been isolated from *G. lucidum* fruiting bodies, mycelium, and spores [31–33]. However, there is limited clinical evidence regarding its efficacy, necessitating systematic reviews to provide consolidated information for healthcare consumers. GLSO, obtained from crushed spores by supercritical CO_2_ extraction, contains predominantly *Ganoderma* triterpenes (GLTs), which are compounds composed of one or more isoprene units and exhibit anti-inflammatory and antitumor activities [25]. There is a burgeoning interest in the immunomodulatory effects of GLSO, with a particular focus on its ability to enhance macrophage phagocytosis [34, 35]. Despite this, the role of GLSO in modulating immune function, especially in mice subjected to psychological stress, remains unexplored, and the mechanisms underlying its potential to promote macrophage phagocytosis are not well understood.

Our study has uncovered that the progression of tumors triggered by psychological stress is exacerbated by the impairment of macrophage phagocytosis, attributed to liposoluble components. Lipidomics analysis has pinpointed LPI (18:0) as a pivotal factor that suppresses macrophage phagocytic capacity under conditions of psychological stress. The present study has demonstrated that GLSO can ameliorate psychological stress-induced tumor progression by bolstering macrophage-mediated phagocytosis of tumor cells *in vivo*. RNA-seq has highlighted that GLSO significantly up-regulates the macrophage Fc gamma R-mediated phagocytosis pathway under stress. Taken together, our findings elucidate that psychological stress-promoted cancer progression is significantly influenced by the inhibition of FcγR phagocytosis signaling by key liposoluble components, such as LPI (18:0). GLSO mitigates the stress-induced suppression of macrophage phagocytosis by modulating the LPI/FcγR-mediated phagocytosis pathway.

## Materials and methods

### Reagents and antibodies

Guangzhou Hanfang Pharmaceutical Co., Ltd., provided GLSO used in this study with quality control data (Lot#: 1004221202), BCA protein assay kit (Cat#: 23227), Alexa Fluor™ 594 anti-Rat (Cat#: A21209), and FluoSpheres carboxyl-modified microspheres (Cat#: F8827) were purchased from ThermoFisher Scientific (USA). The primary antibodies used in this study are anti-F4/80 (Cat#: ab16911, Abcam, Cambridge, UK). Anti-CD16 (Cat#: 80006, Cell Signaling Technology, Inc, USA), Rat monoclonal antibody against CD16/CD32 (Cat #: Ab25235, Abcam, Cambridge, UK), CellTrack^™^ Green CMFDA (Cat#: C2925, Invitrogen, USA), Puromycin (Cat#: 119L0426, Beijing Soleberg Technology Co. Beijing, China), DAPI (Cat#: C1002, Beyotime, Shanghai, China), anti-splenic tyrosine kinase antibody (SYK) (Cat#: 13198 s, Cell Signaling Technology, USA) and anti-phosphorylated spleen tyrosine kinase antibody (p-SYK) (Cat#: 2710 s, Cell Signaling Technology, USA). CID16020046 (Cat#: C288401, Shanghai Aladdin Biochemical Technology Co. Shanghai, China), LPI (18:0) (Cat#: 850104p, Avanti USA, Inc. USA).

### Cell culture

Human peripheral blood mononuclear cells (THP1) were purchased from Ming Zhou Biologicals. Mouse monocyte-macrophage leukaemia cells (RAW 264.7), and mouse ovarian carcinoma epithelial cells (ID8) were purchased from iCell luciferase-labeled. Mouse ovarian epithelial carcinoma cells (ID8-Luc) were purchased from Yuchi Biologicals. THP1 cells were cultured in RPMI 1640 medium containing 10% FBS, 1% penicillin–streptomycin, 1 mM sodium pyruvate, and 0.05 mM mercaptoethanol. RAW 264.7 cells, ID8 cells, and ID8-Luc cells were cultured in a DMEM medium containing 10% FBS and 1% penicillin–streptomycin. ID8-Luc cells are selected for stable luciferase expression by adding 5 μg/ml puromycin approximately every 8 passages.

### Animal care

Female C57BL/6 J mice (4 weeks old) were purchased from Laboratory Animal Center, Southern Medical University (Laboratory Animal Licence No. SCXK (Guangdong) 2021–0041), housed in environmentally controlled conditions with a temperature of 18–22 °C, humidity of 60–70%, and a 12 h light/dark cycle, adaptive feed for at least a week before experiments. All animal experimental protocols in this study adhere to the National Institutes of Health (NIH) Guide for the Care and Use of Laboratory Animals.

**Fig. 1:**
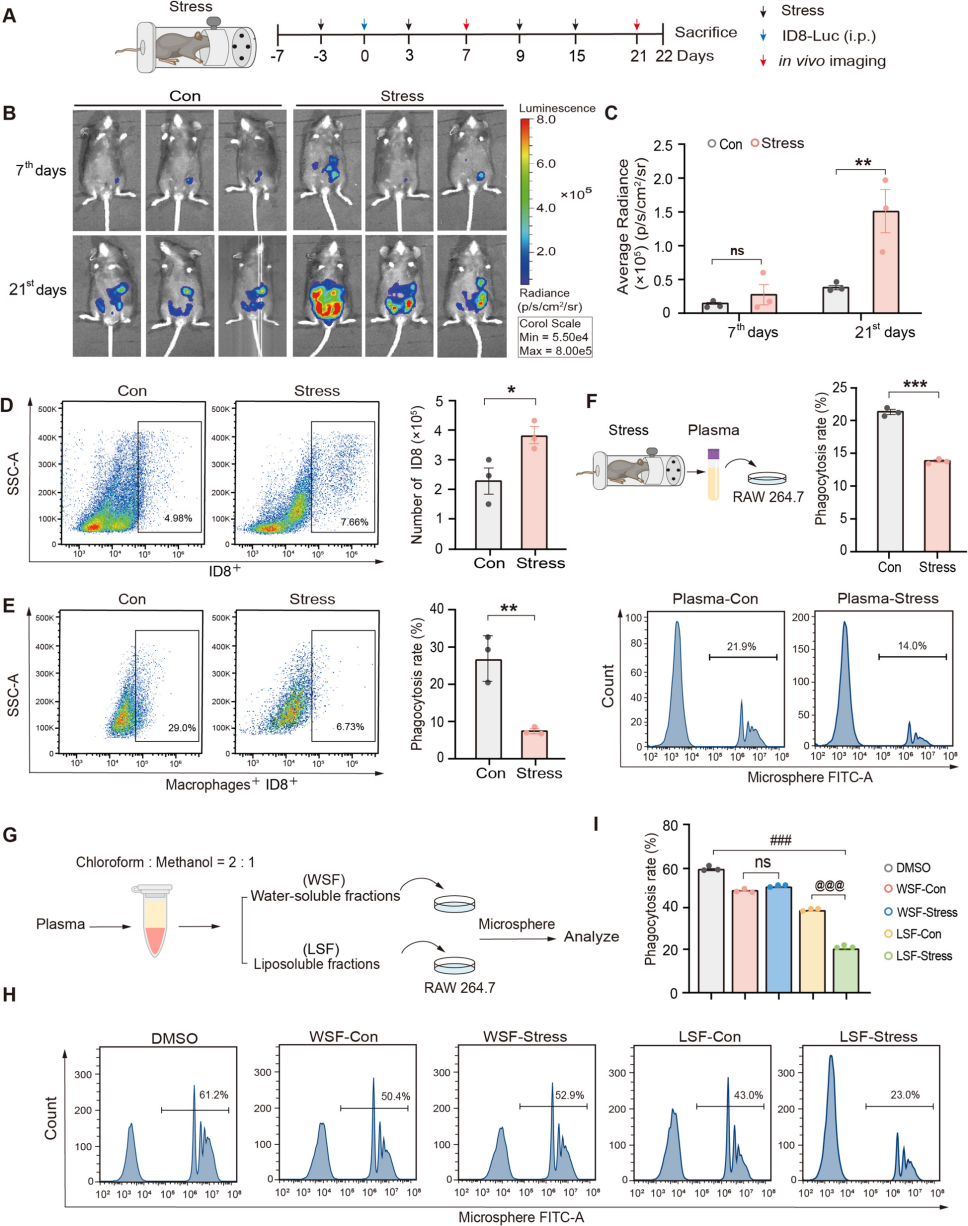
Psychological stress-triggered tumor progression is contributed by liposoluble components impaired macrophage phagocytosis. **A** Schematic of experimental protocol for a mouse ovarian cancer model under restraint stress. **B**, **C** Tumor growth of mice was monitored using an *in vivo* imaging system, with quantification of the average radiant efficiency. **D**, **E** Mice were intraperitoneally injected with CMFDA-labeled ID8 cells, and peritoneal lavage was collected after 24 h. Peritoneal macrophages were labeled with APC anti-mouse F4/80 antibody. Flow cytometry was used to quantify both the tumor cells in the peritoneal cavity of mice and the phagocytosis rate of peritoneal macrophages. **F** Flow cytometric analysis of phagocytic activity against fluorescent microspheres in macrophages pretreated with plasma from restraint-stressed mice. **G** Schematic of screening the effect of water-soluble or liposoluble components in plasma of mice. **H**, **I** RAW 264.7 macrophages were treated with water-soluble or liposoluble fractions of plasma from mice for 4 h and then incubated with fluorescent microspheres for 40 min. The phagocytosis rate was determined by flow cytometry. The values are represented as mean ± SEM (*n* = 3). The *p*-values were determined using unpaired two-tailed Student’s t-test (**D**–**F**, **I**), or one-way ANOVA with LSD (**C**). ^*^P < 0.05,^**^P < 0.01, ^***^P < 0.001 *vs.* Con, ^###^*P* < 0.001 *vs.* DMSO, ^@@@^*P* < 0.001 *vs.* LSF-Con. WSF, water-soluble fractions. LSF, liposoluble fractions

In the first batch of animal experiments, restraint stress was applied for 18 h on the 3^rd^ day before tumor inoculation and the 3^rd^, 9^th^, 15^th^, and 21^st^ day after tumor inoculation. Tumor growth on the 7^th^ and 21^st^ day after inoculation was observed using *in vivo* imaging, respectively (Fig. 1A). In the second batch of animal experiments, mice in the Stress + GLSO-L group and the Stress + GLSO-H group were treated daily with 2.5 g/kg (5 times the effective human dose) and 5 g/kg (10 times the effective human dose) GLSO respectively and subjected to 18 h of restrain stress on the 6^th^ day. The ID8-CMFDA cells were peritoneally injected on the 7^th^ day. Mice were anesthetized with isoflurane on the 8th day, and the peritoneal macrophages and ID8 cells were collected (Fig. 3A). In the third batch of animal experiments, mice in the Stress + GLSO-L group, Stress + GLSO-H group and Stress + LEPT (a recognized immune modulator in clinic, positive control [36, 37]) group were treated daily with 2.5 g/kg GLSO, 5 g/kg GLSO and 16 mg/kg LEPT respectively for 28 days (Fig. 3F). The treatment of restraint stress, tumor inoculation and tumor growth of mice by *in vivo* imaging was as same as the first batch of animal experiments.

### Quantification of ID8 cell number and macrophage phagocytosis rate

Flow cytometry was used to assess the ability of mouse peritoneal macrophages to phagocytose fluorescent microspheres and ID8 ovarian cancer cells. First, fluorescent microspheres were coated with 1% BSA or ID8 cells labeled with CellTracker™ Green CMFDA. The microspheres or labeled ID8-Luc cells were then co-cultured with macrophages at 37 °C for 40 min. After phagocytosis, unphagocytosed fluorescent microspheres or tumor cells were thoroughly washed away with PBS. The remaining cells were collected using a cell scraper, centrifuged at 1000 rpm for 5 min, and the supernatant was discarded. The cells were resuspended in PBS and analyzed using a flow cytometer.

After approximately 8 passages of ID8-Luc cells, 5 μg/mL of puromycin was added to select stable fluorescent clones. C57BL/6 J mice were injected with ID8-Luc (1 × 10^7^ cells/mouse). Following the experimental protocol, the mice were euthanized under isoflurane anesthesia. The peritoneal lavage fluid was centrifuged at 1000 rpm for 10 min at 4 °C, and the supernatant was subsequently removed [38, 39]. In this study, APC anti-rat F4/80 antibody (0.5 μg/mL) was used to label macrophages in the peritoneal lavage fluid [40, 41], and the mixture was incubated at 37 °C for 30 min. After incubation, PBS was added to dilute the antibody, and centrifugation was performed at 1000 rpm for 5 min to remove the supernatant. The cells were resuspended in PBS and analyzed using flow cytometry.

### Western blots analysis

Cells were suspended in RIPA lysis buffer, allowing isolation of total proteins. Then the lysis buffer was centrifuged at 4 °C and 13,000 rpm for 15 min, and cell lysates were used to measure protein concentration using the BCA kit. A protein was incubated with 5 × loading buffer for 10 min at 100 °C. The proteins (20 μg) were then separated on 4–15% SDS–polyacrylamide gel electrophoresis gels and transferred to Immobilon-P polyvinylidene difluoride transfer membranes. These membranes were then blocked with a 5% nonfat milk solution in TBST buffer for 2 h. They were further incubated with primary antibodies, and treated with HRP-conjugated anti-rabbit or anti-mouse secondary antibodies. Protein expression was assessed visually using the FDbio-Pico ECL Kit (Fude Biological Technology, China) and imaged using the Tanon 5200 Chemiluminescent Imaging System (Tanon, China). The pixel intensity density of the band was measured using Image J software and was then normalized to the equivalent strain control intensity.

### RNA sequencing analysis

Following the complete lysis of the macrophage samples in 1 ml of TRIzol reagent, the entire TRIzol solution was transferred to a cryovial that was free of RNase contamination. Library preparation and sequencing were performed by BGI Gene Company (Shenzhen, China). Differential gene expression analysis was performed using DESeq software (version: 1.20.0) to compare gene expression levels between two experimental groups. The genes with low expression across all samples (FPKM < 0.5 in all samples) were removed, and the genes with adjusted *P* value below 0.05 were extracted as significantly differentially expressed genes. The differentially expressed genes were subjected to Gene Ontology (GO) and Kyoto Encyclopedia of Genes and Genomes (KEGG) enrichment analysis using the tool available at http://www.bioinformatics.com.cn/. GO categories and KEGG pathways were considered for analysis with *P* < 0.05.

### Quantitative polymerase chain reaction (qPCR) analysis

Total RNA from macrophages in the sample was extracted using a Trizol reagent. *FCGR3* was amplified from oligo(dT)-primed cDNA by 25 PCR cycles (40 s at 95 °C, 40 s at 57 °C, and 55 s at 72 °C). The PCR reaction was carried out on a CFX connect amplifier Real-Time PCR Detection System (Bio-Rad, USA), and after amplification, melting curve analysis was performed to determine the specificity of the amplified products. The primer sequences used in this study are as follows: *GAPDH* forward, CTTCACCACCATGGAGAAGGC; *GAPDH* reverse, GGCATGGACTGTGGTCATGAG; *FCGR3* forward, TACAGGGTGCTCGAGAAGGA,* FCGR3* reverse, GGGTGGGGAGAGGTTTGTCTGG.

### Phospholipids extraction and analysis

Each plasma sample was placed into separate glass tubes, and then a pre-prepared solution of chloroform/methanol (2:1, v/v) was added. After thorough vortex mixing, the tube was placed on ice for 1 h and vortexed vigorously every 10 min to ensure complete extraction. The mixture was then centrifuged at 1000 rpm for 5 min. The centrifuged solution was separated into three layers, with the organic phase collected from the bottom layer using a funnel and filter paper previously soaked in a chloroform–methanol mixture. The resulting clarified liquid was placed on ice and dried under nitrogen gas. The dried residue was reconstituted in 200 μL of mass spectrometry-grade methanol, transferred to a 1.5 mL EP tube, and centrifuged at 4 °C and 14,000 rpm for 30 min. The supernatant was transferred to a new 1.5 mL EP tube and stored at − 80 °C.

### Plasma phospholipidomics by LC–MS/MS

Take 20 nmol Pi samples, place them in a brown liquid phase vial with an inner tube, make up the volume to 40 μL with methanol, and then add 2 μL of internal standard to each sample to ensure that there is no precipitation and no bubbles. The samples should be detected using LC–MS/MS. Detection mode: positive/negative ions, mass range: 200–1800 Da. Liquid phase analysis conditions: Column: HSST3 column; Column temperature: 40 ℃. Phase A, water: acetonitrile = 1:1; Phase B, Isopropanol: acetonitrile = 9:1, both containing 10 mM ammonium formate and 0.01% ammonia. The solvent was developed with a flow rate at 0.3 mL/min by the following gradient elution program: 0–5 min, 30–43% B; 5.1–14.0 min, 50–70% B; 14.1–24 min, 70–99% B; 24.1–28 min, 30% B. Injection volume: 2 µL. Mass spectrometry analysis conditions: Source gas parameters: Ion source: Electrospray ionization source (ESI spray voltage: positive ions, 3.0 kV, negative ions, − 2.8 kV) S-lens RF level: 65 Capillary temperature: 350 °C. Auxiliary gas heater temperature: 320 °C Sheath gas: 30 Arb Auxiliary gas: 15 Arb Recoil gas: 0 Arb Mass spectrometry acquisition parameters: Acquisition mode: Full MS-dd-MS2 Full MS: Resolution 70,000; AGC, 3e6; MIT, 100 ms dd-MS2: resolution, 17,500; AGC, 1e5; WITH, 50 ms.

### Immunofluorescence analysis

The differentiation of THP-1 cells (a human monocytic leukemia cell line) into macrophages is a standardized in vitro model. THP-1 cells were treated with phorbol 12-myristate 13-acetate (PMA) at concentrations of 100–200 nM for 48 h, followed by incubation in PMA-free medium for 24–48 h [42, 43]. THP1 cells were placed in confocal culture dishes and induced to differentiate into macrophages. 1 mL of 4% paraformaldehyde was added to fix the macrophages at room temperature for 10 min, followed by a 5 min wash with PBS. Cells were then permeabilized with Triton X-100 for 10 min. The cells were blocked with goat serum for 1 h and then incubated with anti-CD16/CD32 antibody (1:100) overnight at 4 °C. The next day, the primary antibody was removed, and the cells were washed with PBS for 5 min. Cells were incubated with Alexa Fluor™ 594 anti-Rat antibody (1:500) at room temperature for 2 h. Finally, DAPI (1:1,000) was added for 15 min. Fluorescence microanalysis was performed using a SpinSR laser scanning confocal microscope system (Olympus, Japan). Image acquisition and merging were performed using Olyvia 4.1 software.

### Statistical analysis

Statistical analysis was carried out using GraphPad Prism 8 software and SPSS 25.0. All the data were presented as the mean values ± standard error of the mean (SEM). For comparison between the two groups, an unpaired two-tailed Student’s t-test was used, and a significant difference was considered when *P* < 0.05. One-way ANOVA was used to compare differences between three or more groups, the LSD (Least Significant Difference) test was employed for comparisons made solely with the control group; the Tukey test was utilized for analyses requiring multiple comparisons. A significant difference was considered if *P* < 0.05.

## Results

### Psychological stress-triggered tumor progression is contributed by liposoluble components impaired macrophage phagocytosis

In this study, we first established ID8 ovarian cancer in mice to explore the critical components regulating macrophage phagocytosis in psychological stress-promoted tumor progression. Mice received 18 h restraint stress weekly for 28 days, and were inoculated with ID8-Luc ovarian cancer cells on the 3^rd^ day after stress (Fig. 1A). Tumor growth of mice was monitored using an *in vivo* imaging system on days 7^th^ and 21^st^ after tumor inoculation. As depicted in Fig. 1B and C, stress significantly promoted cancer progression, which is consistent with our previous study. In addition, peritoneal macrophages were identified using APC anti-Rat F4/80 antibody labeling. Compared to the control group, the number of tumor cells in the peritoneal lavage fluid was significantly increased in stress group (Fig. 1D). Further, phagocytosis of tumor cells by peritoneal macrophages in mice was significantly reduced in the stress group (Fig. 1E). Besides, the plasma from mice treated with or without a daily 18 h of restraint stress were applied in RAW 264.7 macrophages (Fig. 1F), and it is found that results from above *in vitro* is in a pattern similar with *in vivo* experiments. To investigate the critical components affecting macrophage phagocytosis during psychological stress, the water-soluble components and liposoluble components from plasma were isolated by the Folch method (Fig. 1G). Interestingly, compared with the control plasma of mice, the liposoluble fractions from plasma in mice subjected to restraint stress significantly inhibited macrophage phagocytosis, while the water-soluble fractions had no significant effect on macrophage phagocytosis (Fig. 1H, I). These results suggest that psychological stress-mediated phagocytosis inhibition of macrophage-promoted tumor growth is associated with the liposoluble components.

### LPI is identified as a key component that suppresses macrophage phagocytosis under stress

**Fig. 2:**
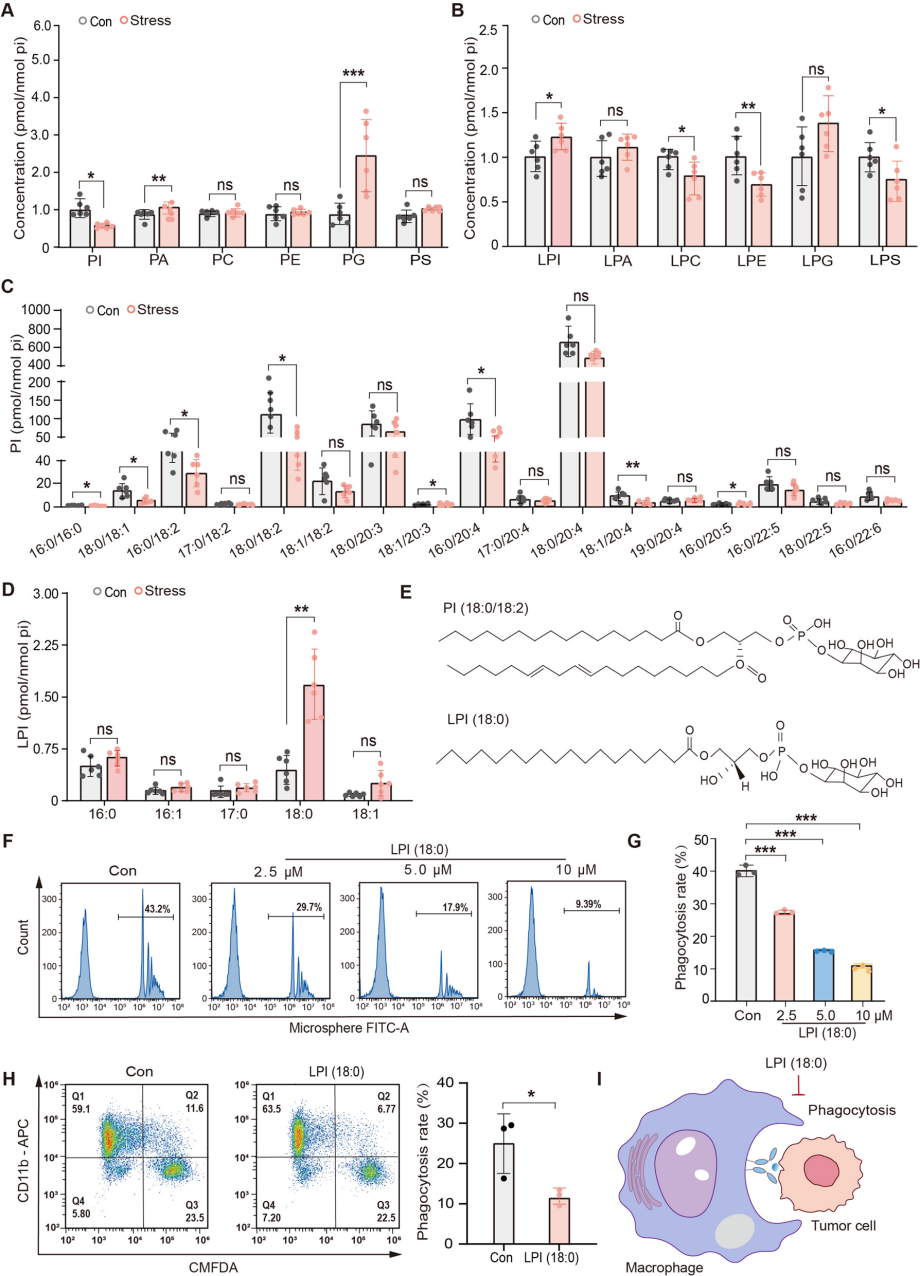
LPI is identified as a key component suppressing macrophage phagocytosis under stress. **A**, **B** Relative concentration of phospholipids and lysophospholipids. **C**, **D** MS2 analysis of PI from plasma of restraint stress mice (*n* = 6). **E** Chemical structural formulas of PI(18:0/18:2) and LPI(18:0). **F**, **G** THP1 macrophages treated with LPI (18:0) at different concentrations were incubated with fluorescent microspheres for 40 min and the phagocytosis rate was determined by flow cytometry (*n* = 3). **H** The phagocytic activity of LPI (18:0)-treated THP1 macrophages (10 μM, 4 h) to tumor cells was assessed by flow cytometry (*n* = 3). **I** Schematic diagram illustrating that LPI inhibits the phagocytic function of macrophages. The values are represented as mean ± SEM. The *p*-values were determined using unpaired two-tailed Student’s t-test (**A**–**D**, **H**), or one-way ANOVA with LSD (**G**). The significant difference was marked as ^*^P < 0.05, ^**^P < 0.01, ^***^P < 0.001 *vs.* Con. Each dot represents a single phospholipid or lysophospholipid. PI, phosphatidylinositols. PA, phosphatidic acid. PC, Phosphatidylcholine. PE, Phosphatidylethanolamine. PG, Phosphatidylglycerin. PS, Phosphatidylserine. LPI, lysophosphatidylinositols. LPA, lysophosphatidic acid. LPC, lysophosphatidylcholine. LPE, lysophosphatidylethanolamine. LPG, lysophosphatidylglycerol. LPS, lysophosphatidylserine

To identify the specific liposoluble compound that affects macrophage phagocytosis, the content and composition of liposoluble compounds in the plasma of restraint-stressed mice were analyzed by LC–MS/MS-based phospholipidomics. Results as depicted in Fig. 2A and B, we observed a notable dysregulation in plasma phospholipid metabolites among mice exposed to restraint stress. Specifically, a significant decrease in PI levels and a corresponding increase in LPI levels were detected compared to the control group. This pattern suggests a potential metabolic conversion between PI and LPI under stress conditions, which piqued our interest. Moreover, phosphatidylinositol (PI) is known to play a pivotal role in orchestrating phagocytosis through the precise spatiotemporal regulation of its metabolic network [17, 44]. Further mass spectrometric analysis of the PI class of phospholipids revealed a marked increase in LPI (18:0) content in the plasma of mice subjected to restraint stress, accompanied by a variable decrease in native PI levels (Fig. 2C, D). The chemical structural formulas of PI (18:0/18:2) and LPI (18:0) are shown in Fig. 2E. LPI, recognized as a bioactive lipid, is implicated in the pathophysiology of various diseases [45]. To determine whether LPI serves as a critical mediator in the inhibition of macrophage phagocytic function, the phagocytosis of LPI (18:0)-treated macrophages was detected *in vitro* by flow cytometry[42, 46]. We found that LPI (18:0) inhibits macrophage phagocytosis of fluorescent microspheres in a dose-dependent manner (Fig. 2F, G). In addition, LPI (18:0) significantly suppressed the phagocytosis of macrophages to tumor cells (Fig. 2H). Taken together, as depicted in Fig. 2I, these results suggest that LPI (18:0) can significantly inhibit the phagocytic capacity of macrophages against tumor cells and fluorescent microspheres *in vitro*, although the underlying mechanism requires further investigation.

### GLSO inhibits ovarian cancer progression by enhancing macrophage phagocytic activity

**Fig. 3:**
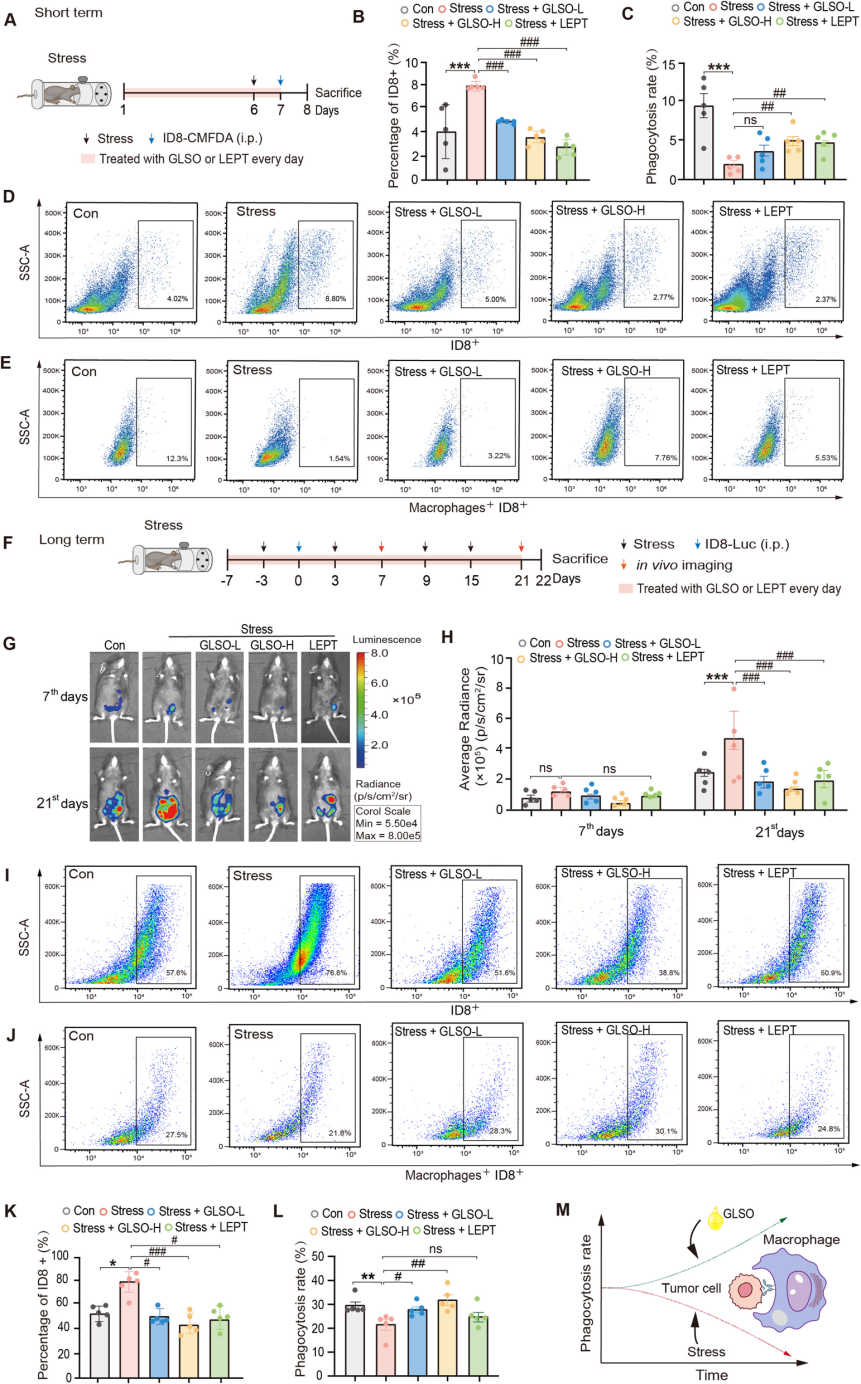
GLSO alleviates tumor progression by enhancing the phagocytic function of macrophages. **A** Schematic of short-term experimental design. **B**, **D** The percentage of ID8 cells in the abdominal cavity of mice was detected using flow cytometry. **C**, **E** The phagocytosis rate of peritoneal macrophages in mice was assessed by flow cytometry. **F** Schematic of long-term experimental design. **G**, **H** Tumor growth of mice was monitored using an in vivo imaging system, with quantification of the average radiant efficiency. **I**, **K** The percentage of ID8 cells in mice was assessed on the 21^st^ day after tumor inoculation. **J**, **L** Phagocytosis of mouse peritoneal macrophages was assessed on the 21^st^ day after tumor inoculation. **M** Schematic diagram illustrating the effect of GLSO on mitigating stress-induced impairment of macrophage phagocytosis. Data are presented as mean ± SEM (*n* = 5). *P* value was determined using Tukey one-way ANOVA. Significant difference was marked as, ^*^P < 0.05, ^**^P < 0.01, ^***^P < 0.001 *vs.* Con, ^#^*P* < 0.05, ^##^*P* < 0.01, ^###^*P* < 0.001 *vs.* Stress

GLSO has been widely demonstrated to exhibit immune-enhancing properties [25, 47]. To examine whether GLSO can alleviate the inhibitory effect of LPI (18:0) on macrophage phagocytosis, short- and long-term immunosuppression models induced by restraint stress were established (Fig. 3A, F). In the short-term model (Fig. 3B–E), the stress group displayed a significant increase in the percentage of tumor cells within the abdominal cavity. However, pretreatment with high-dose GLSO significantly reduced the tumor cell percentage in stressed mice. Furthermore, the phagocytic activity of peritoneal macrophages was reduced in the stress group. In contrast, mice receiving high-dose GLSO exhibited a significant increase in macrophage phagocytic activity compared to the stress group. In the long-term stress model, an* in vivo* imaging system was used to monitor tumor progression on the 7^th^ and 21^st^ days post-tumor inoculation. Stress significantly accelerated cancer progression; however, GLSO effectively suppressed ovarian cancer advancement in stressed mice (Fig. 3G, H). As the tumor grows, a significantly higher percentage of tumor cells was observed in the stress group compared to the control group. GLSO pretreatment significantly decreased the number of tumor cells in mice subjected to restraint stress, demonstrating its tumor growth-inhibitory effects (Fig. 3I, K). Furthermore, compared with the control group, the phagocytosis rate of peritoneal macrophages was significantly decreased in mice subjected to restraint stress (Fig. 3J, L). In contrast, GLSO pretreatment effectively improved the macrophage phagocytosis compared to the stress group. Taken together, these findings indicate that GLSO alleviates stress-induced tumor progression by improving the phagocytosis of macrophages to tumor cells (Fig. 3M).

### FcγR serves as a critical target for GLSO in mitigating stress-repressed macrophage phagocytic function

**Fig. 4:**
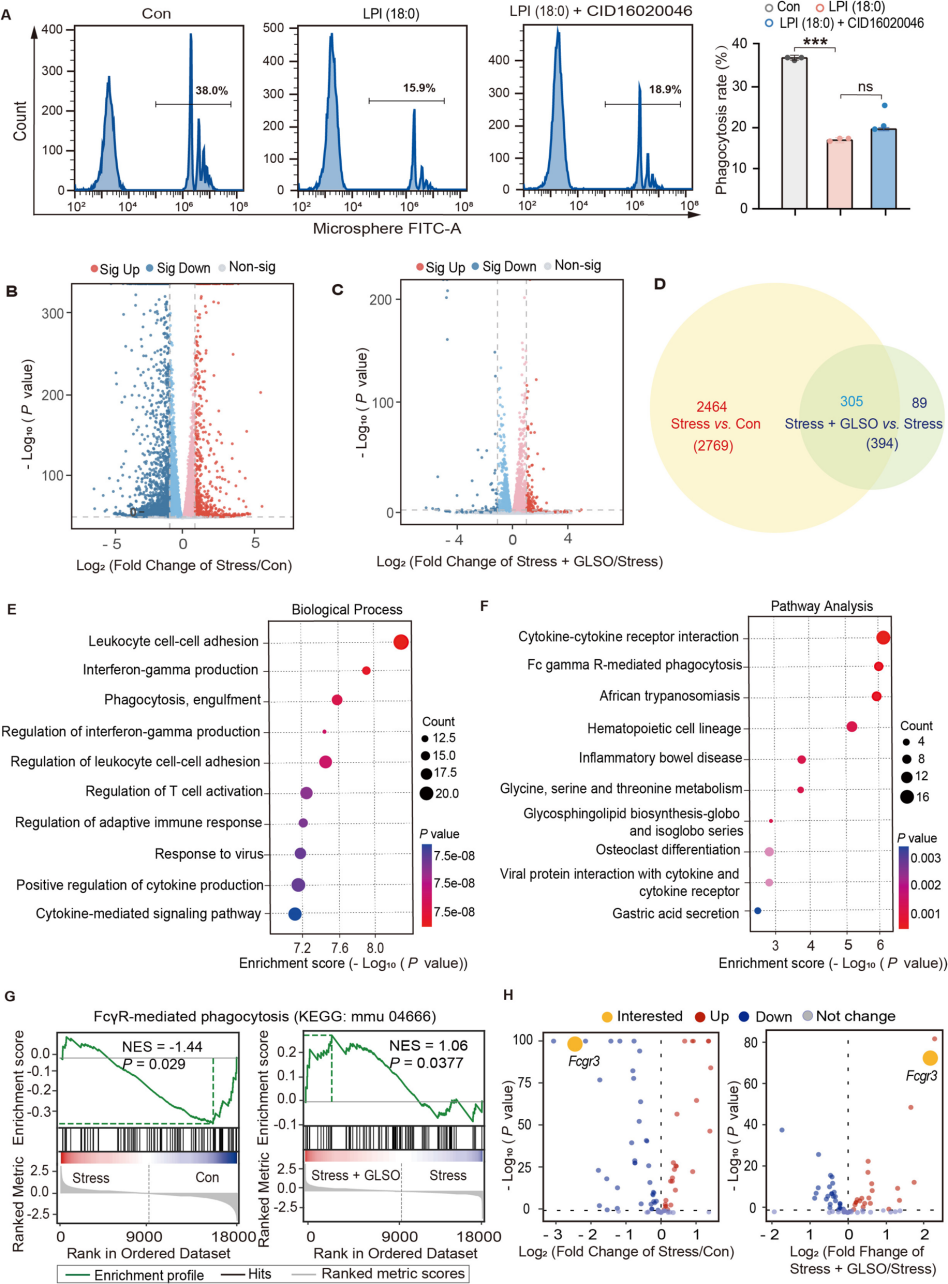
FcγR serves as a critical target for GLSO in mitigating stress-induced suppression of phagocytic function. **A** The phagocytosis rate of macrophages treated with LPI or CID16020046 was assessed. Data are presented as mean ± SEM (*n* = 3). The *p*-values were determined using Tukey one-way ANOVA, ^***^P < 0.001 *vs.* Con. **B**, **C** Volcano map (Stress *vs.* Control, Stress + GLSO *vs.* Stress) of differentially expressed genes (*n* = 5). **D** Venn diagrams showing differential and overlapping genes identified by RNA-seq from (Stress *vs.* Control) and (Stress + GLSO *vs.* Stress). **E** The overlapping 305 differential genes were analyzed for Gene Ontology (GO) bioprocess enrichment. **F** The Kyoto Encyclopedia of Genes and Genomes (KEGG) pathway analysis of 305 overlapping differentially expressed genes associated with phagocytosis. **G**, **H** The core genes involved in the Fc pathway-mediated phagocytic pathway (GSEA) and core genes are shown in a volcano diagram

G protein-coupled receptor 55 (GPR55), recognized as the receptor for LPI, is highly expressed in immune cells [48]. Upon activation, GPR55 stimulates downstream signaling pathways, including p38 and ERK1/2 [49]. To investigate whether LPI (18:0) exerts its inhibitory effect on phagocytosis through GPR55, we co-stimulated cells with LPI (18:0) and the GPR55 receptor antagonist CID16020046. As depicted in Fig. 4A, LPI (18:0) significantly impaired the phagocytic function of THP1 macrophages compared to the control group, while the GPR55 inhibitor failed to alleviate the phagocytic inhibition induced by LPI (18:0). To further elucidate the suppressive effects of stress on macrophage phagocytosis and the protective mechanism of GLSO, RNA-seq analysis on macrophages from mice subjected to stress and GLSO treatment was conducted. The volcano plot illustrated that 1949 upregulated differentially expressed genes (DEG) and 820 downregulated DEG were identified between stress group and control group (Fig. 4B), while 295 upregulated DEG and 99 downregulated DEG were shown in GLSO group when compared to stress group (Fig. 4C). Venn diagrams analysis further revealed that 305 overlapping DEG were identified between (stress *vs.* Con) and (stress *vs.* GLSO + stress) (Fig. 4D).

Subsequently, Gene Ontology (GO) term enrichment analysis revealed that these overlapping 305 differential genes were involved in various biological functions and processes. The most significantly enriched biological processes included Leukocyte cell–cell adhesion, Interferon-gamma production, and Phagocytosis, engulfment (Fig. 4E). Besides, Kyoto Encyclopedia of Genes and Genomes (KEGG) enrichment analyses of these 305 differential genes were performed and found that these genes are involved in the Fc gamma R-mediated phagocytosis pathway (Fig. 4F). Employing Gene Set Enrichment Analysis (GSEA), the molecular pathways critical for macrophage phagocytosis were identified, pinpointing core genes that influence phagocytic function. As depicted in Fig. 4G and H, the phagocytosis-related pathway is Fc gamma R-mediated phagocytosis (KEGG: mmu04666). Notably, the results showed that among the signaling pathways regulating phagocytosis, the FcγR gene had the most significant changes. Stress treatment downregulated *Fcgr3* expression, while GLSO administration reversed this effect. In summary, we propose that stress may diminish Fc gamma R-mediated phagocytosis, while GLSO may ameliorate this suppression of phagocytic activity.

### GLSO alleviates LPI/FcγRIII-mediated inhibition of macrophage phagocytosis

**Fig. 5:**
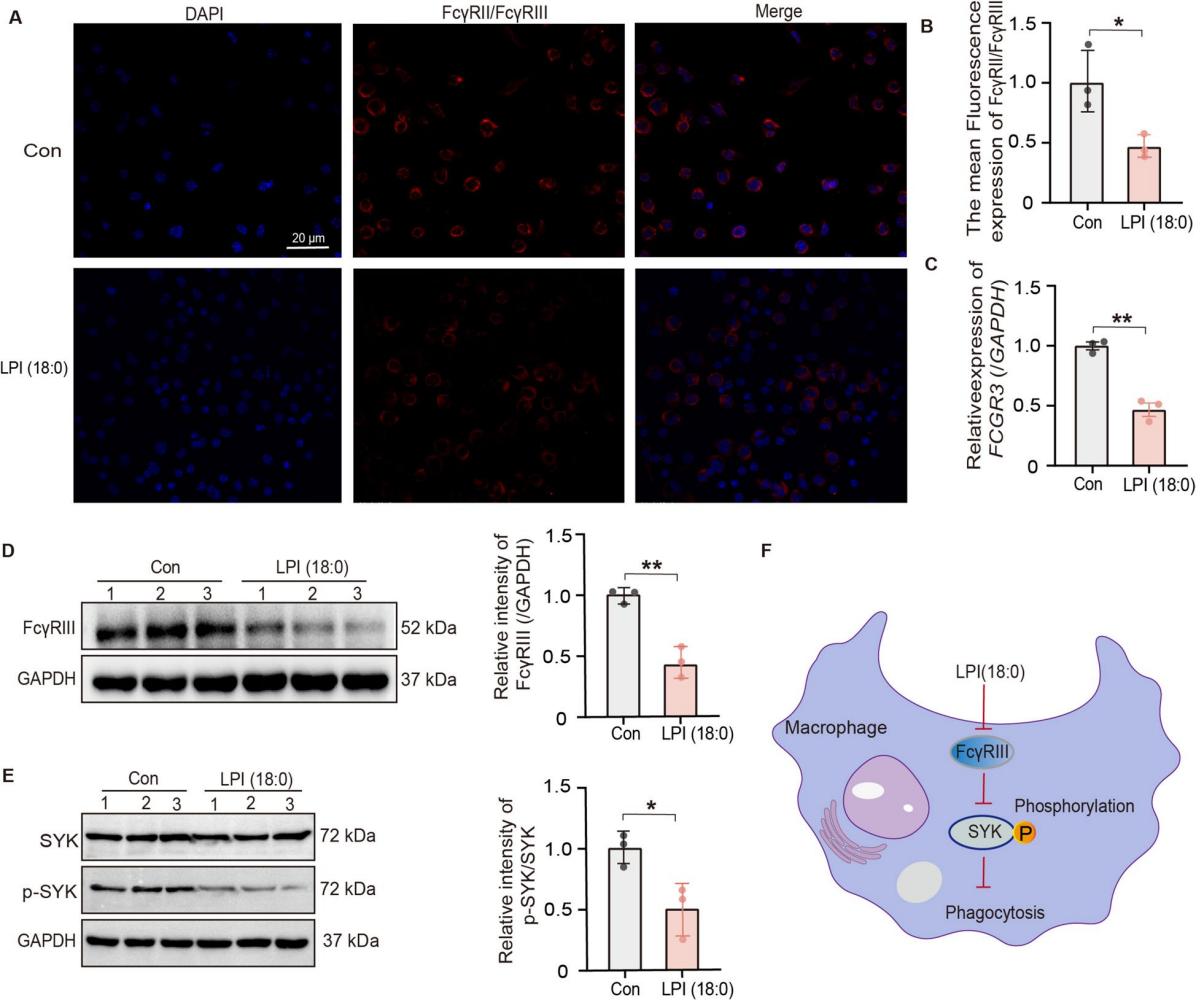
LPI mediated the suppression of the FcγRIII/SYK/p-SYK phagocytic signaling pathway in macrophages. **A**, **B** THP1 macrophages were treated with LPI (18:0) (10 μM, 18 h). Anti-CD16/CD32 antibodies labeled FcγRIII/FcγRII, and laser confocal was detect, and mean fluorescence quantification of protein expression of FcγRII/FcγRIII. **C** The mRNA expression of *FCGR3* in LPI (18:0)-treated THP1 macrophages was detected. **D**, **E** The FcγRIII, SYK, and p-SYK protein expression in LPI (18:0)-treated THP1 macrophages was detected. **F** LPI (18:0) mediated the suppression of the FcγRIII/SYK/p-SYK phagocytic signaling pathway in macrophages. Data are presented as mean ± SEM (*n* = 3). The *p*-values were determined using an unpaired two-tailed Student’s t-test (**B**–**E**). A significant difference was marked as ^*^P < 0.05, ^**^P < 0.01 *vs.* Con

As depicted in Fig. 5A and B, LPI (18:0) significantly inhibited FcγRII/FcγRIII expression in macrophages, and the gene of *FCGR3* was significantly reduced (Fig. 5C). The FcγRIII protein expression was reduced after the addition of LPI (18:0) compared with the control group (Fig. 5D). Similarly, the same trend was observed for SYK phosphorylated proteins, with a decrease in the level of SYK phosphorylation after the addition of LPI (18:0) compared to the control group (Fig. 5E). To summarize, as shown in Fig. 5F, LPI (180:0) mediated the suppression of the FcγRIII/SYK/p-SYK phagocytic signaling pathway in macrophages.

**Fig. 6:**
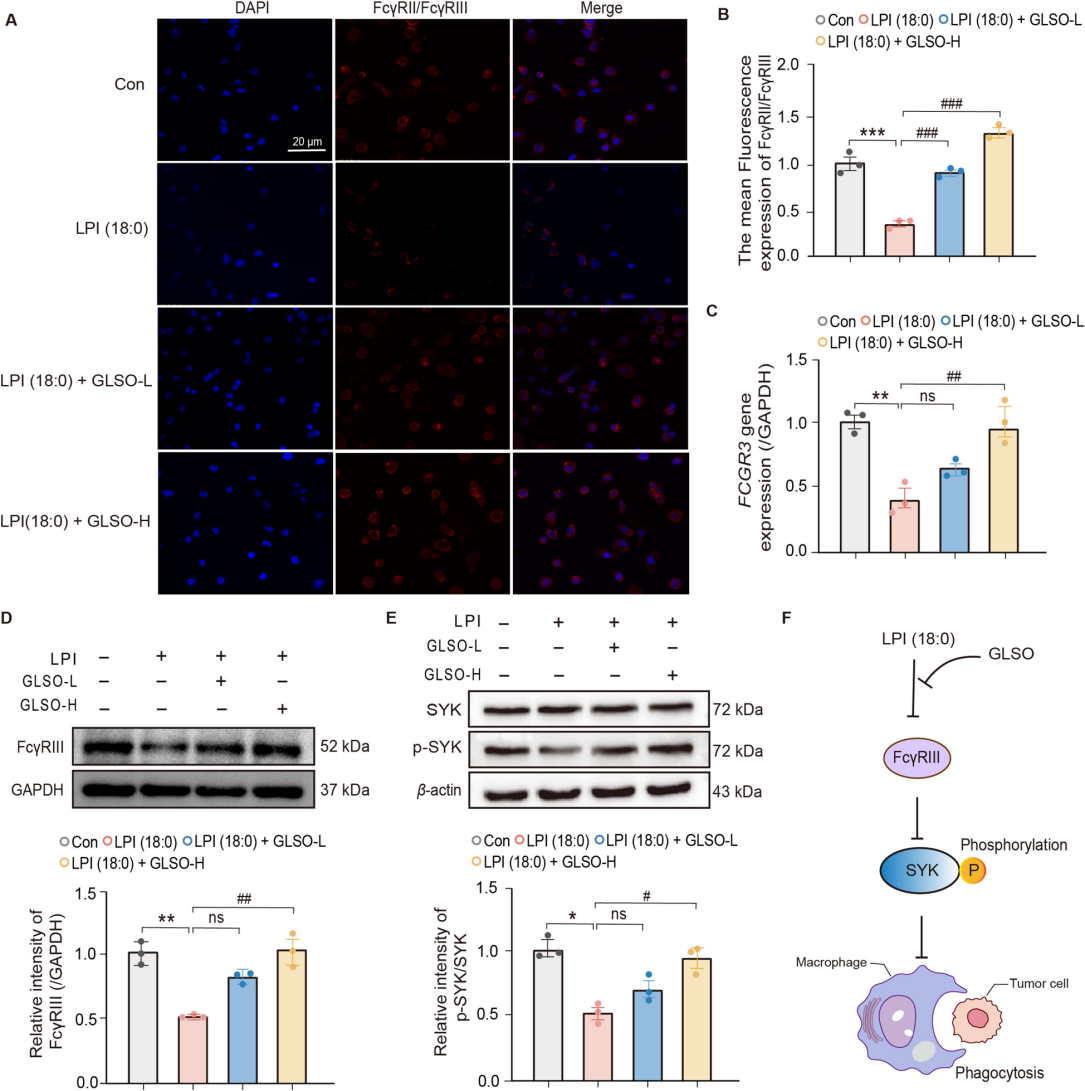
GLSO mitigates the LPI-mediated suppression of the FcγRIII/SYK/p-SYK phagocytic signaling pathway. **A**, **B** THP1 cells were treated with PMA (200 nM, 2 d), followed by pre-protection with GLSO (0.05, 0.1 mg/ml, 24 h) and finally LPI (18:0) (10 μM, 18 h). Anti-CD16/CD32 antibodies labeled FcγRIII/FcγRII, and laser confocal was detect, and mean fluorescence quantification of protein expression of FcγRII/FcγRIII. **C** The mRNA levels of *FCGR3* in THP1 macrophages with or without GLSO treatment. **D**, **E** The protein expression of FcγRIII, SYK, and p-SYK in THP1 macrophages with or without GLSO treatment was detected. **F** GLSO alleviates the phagocytic inhibition of the SYK/p-SYK axis in macrophages mediated by LPI (18:0). Data are presented as mean ± SEM (*n* = 3). The *p*-values were determined using Tukey one-way ANOVA. Significant difference was marked as ^*^P < 0.05, ^**^P < 0.01, ^***^P < 0.001 *vs*. Con, ^#^*P* < 0.05, ^##^*P* < 0.01, ^###^*P* < 0.001 *vs.* LPI

To assess the efficacy of GLSO by alleviating LPI-mediated inhibition of FcγRIII/SYK/pSYK phagocytosis signaling, we pre-protected THP1 macrophages by administering GLSO in advance. The results depicted in Fig. 6A and B show that GLSO significantly alleviated the LPI-induced suppression of FcγRII/FcγRIII in THP1 macrophages. Furthermore, GLSO alleviated the inhibition of FcγRIII caused by LPI (18:0). The mRNA expression of *FCGR3* was significantly downregulated in the LPI group compared to the control group. Yet, GLSO-H mitigated the reduction of *FCGR3* mRNA expression induced by LPI (18:0) (Fig. 6C). Similarly, the expression of FcγRIII protein was consistent with the result of mRNA expression (Fig. 6D), while there was no significant trend in SYK expression, the level of p-SYK was significantly reduced in the LPI (18:0) group compared to the control group. As well as GLSO was also able to alleviate the phosphorylation level of SYK (Fig. 6E). In conclusion, as depicted in Fig. 6F, the LPI-mediated FcγR phagocytosis pathway is identified as a crucial axis underlying the therapeutic effect of GLSO under psychological stress.

## Discussion

In recent years, numerous experimental studies have accumulated convincing evidence that phospholipid composition plays an important role in macrophage phagocytosis, and some phospholipid components can directly act as signaling molecules involved in the transduction and activation of signaling pathways [50, 51]. Nevertheless, the connection between psychological stress and phospholipid metabolism still needs to be clarified. Our preliminary research results and existing literature suggest that psychological stress and abnormal phospholipid metabolism are closely linked to the occurrence of various diseases [52, 53]. In addition, significantly increased levels of LPI, LPG, and LPS were found in tumor tissues from patients with colorectal cancer [54], while increased levels of LPI, LPA, and LPC were observed in the plasma of ovarian cancer patients [55]. However, the regulatory mechanisms of lysophosphatidylinositol in the tumor microenvironment are not fully understood.

Based on our observations of *in vivo* imaging of tumors in mice, we confirmed that psychological stress exacerbates the growth and metastasis of ovarian cancer in mice, and our findings are consistent with numerous previously published studies [52, 56, 57]. Notably, psychological stress can disrupt cellular metabolism and lead to increased lipid compounds in plasma [54, 58]. The increase in lipids in the plasma of stressed mice observed in our study supports exactly this view. The observation that plasma from the control group suppresses macrophage phagocytosis, as compared to the DMSO group, could potentially be attributed to the complex and undefined components present in the mouse plasma. There is limited literature directly addressing the inhibitory effects of normal mouse plasma on macrophage phagocytosis. Our focus in this study is to compare the effects of stress on components (water-soluble or lipid-soluble) that may influence macrophage phagocytosis. Interestingly, we found that the liposoluble components from plasma in mice subjected to restraint stress significantly inhibited macrophage phagocytosis. Lipid composition may find a breakthrough in revealing the mechanisms that inhibit the reduction of phagocytosis in macrophages [44]. In this research, the plasma from stressed mice using LC–MS/MS revealed that LPI is the most altered phospholipid component before and after restraint stress. Secondary mass spectrometry analysis identified LPI (18:0) as the most abundant LPI species. Given the critical role of PI in orchestrating phagocytosis through its metabolic network and its involvement in pathways such as PI3K/PIP3 and VPS34/PI3P [44, 59], we propose that LPI (18:0) might be a key substance impairing the phagocytosis function of macrophages. Subsequent in vitro experiments confirmed that the exogenous addition of LPI (18:0) significantly inhibits the phagocytic function of macrophages as demonstrated by flow cytometry results. Certainly, we need more work next to characterize how LPI (18:0) regulates phagocytosis in macrophages.

As is well known, GLSO mainly contains triterpenes and lipid components such as fatty acids and shows excellent antitumor activity both *in vitro* and *in vivo* [60, 61]. In this study, GLSO can improve the phagocytic function of macrophages in mice with psychological stress, thereby inhibiting the progression of ovarian cancer in mice. Jin et al. research found that GLSO possesses significant immunomodulatory properties [24], which is consistent with our findings. Furthermore, the results of tumor cells and macrophage phagocytosis rates in the peritoneal lavage fluid of mice confirmed the role of GLSO in promoting macrophage phagocytic function. To elucidate the intrinsic relationship between stress-induced impairment of macrophage phagocytic function and the intervention effects of GLSO, we conducted RNA-seq analysis of mouse macrophages. Bioinformatic analyses (GO/KEGG) revealed significant enrichment in phagocytosis-related pathways (FcγR-mediated phagocytosis, engulfment), immune signaling (cytokine interactions), and cellular communication (leukocyte adhesion), and reported in numerous researches [21, 62–64]. Much research has demonstrated that FcγR-mediated phagocytosis is one of the most direct and effective immune clearance mechanisms in the immune system, playing a crucial role in host defense [65–67]. Hence, it would be interesting to investigate how to enhance macrophage FcγR expression or activate Fcγ receptors to increase their phagocytic capacity. Furthermore, the regulatory mechanisms by which GLSO enhances macrophage phagocytic function also warrant further investigation.

Typical phagocytic receptors, including FcγR, CR3, TLR, and other aggregates, improve the biophysical properties of macrophage membranes [23, 68]. *FCGR* genes were all demonstrated to be associated with various cancer types or drug responses [69, 70]. Xu confirmed that *FCGR3A* had highly significant positive associations with *FCGR1A* in various cancers [71]. This evidence may help us infer the correlation between *FCGR* and cancers. In our research, we identified significant enrichment of the Fcγ receptor family, *Fcgr1* (CD64 or FcγRI), *Fcgr2* (CD32 or FcγRII), *Fcgr3* (CD16 or FcγRIII), *Fcgr4* (CD16-2 or FcγRIV), and so on, in the genes associated with phagocytosis-related pathways as indicated by GO and KEGG enrichment analyses. Importantly, GLSO significantly enhances the expression of *Fcgr3*, which aligns with the observation that GLSO mitigates the stress-induced impairment of macrophage phagocytic function. Among this family of receptors, Nemoto and his colleagues reported that increased expression of *Fcgr3* improves phagocytosis in macrophages [72]. FcγRIII (CD16) is typically known for its role in antibody-dependent cellular phagocytosis through interactions with the Fc region of antibodies. However, recent studies have shown that FcγRIII can also enhance phagocytosis in an antibody-independent manner through direct interactions with tumor, pathogens, or BSA-opsonized beads [73–75]. Our study yielded interesting findings, though some limitations should be acknowledged. Firstly, our results were derived from *in vitro* experiments using THP1 cells co-cultured with LPI (18:0); further studies are needed to examine LPI (18:0)'s regulatory effects on Fcgr3 gene expression in primary macrophages. Secondly, we did not investigate LPI (18:0)'s impact on the expression of other FcγR family members, including Fcgr1, Fcgr2 and Fcgr4. Additionally, the mechanisms underlying GLSO's ability to alleviate stress-induced inhibition of FcγR expression by LPI (18:0) remain to be elucidated.

Notably, our results reveal that GLSO could alleviate the inhibition of *Fcgr3* caused by LPI (18:0), and the expression of *Fcgr3* was significantly down-regulated. Similarly, the expression of the FcγRIII protein was consistent with the results of the gene, and GLSO was able to alleviate the phosphorylation level of SYK. Research has shown that the activation of SYK family proteins can trigger the transmission of downstream phagocytic signaling, including the transcription and translation of FcγR genes [20, 67]. FcγRIII (CD16) mediates both antibody-dependent phagocytosis and antibody-independent immune recognition. As noted, it directly binds tumor antigens, microbial PAMPs, and opsonized BSA beads to enhance phagocytosis. We first demonstrate GLSO upregulates macrophage FcγRIII expression *in vitro* and *in vivo*. This elevation promotes direct macrophage-tumor cell interactions, boosting tumor cell recognition and phagocytosis. In conclusion, our findings demonstrate that GLSO enhances macrophage phagocytic function through potential activation of the FcγR signaling pathway, leading to upregulated FcγR expression, thereby facilitating phagocytosis. RNA-seq analysis revealed that GLSO has the  most pronounced effect on the expression of the *Fcgr3* in macrophages. although we did not fully elucidate the mechanisms by which GLSO regulates *Fcgr3* expression in this study. Future studies could employ *Fcgr3* promoter assays to confirm how GLSO promotes the transcriptional activation of *Fcgr3*.

## Conclusion

LPI is a key substance produced under psychological stress and impairs the body's immunity. LPI inhibits macrophage phagocytosis by affecting the activation of macrophage phagocytosis signals, resulting in low immunity and the growth and deterioration of tumors due to premature elimination of tumor cells. The present study verified the protective effect of GLSO on psychological stress-evoked tumor progression, suggesting that its mechanism of action may involve the LPI-mediated FcγR phagocytic pathway.

## Data Availability

The datasets are available from the corresponding author upon reasonable request.

## Abbreviations

FcγR: Fcγ receptor
GLSO: *Ganoderma lucidum* spore oil
GO: Gene ontology
KEGG: Kyoto Encyclopedia of Genes and Genomes
LEPT: Lentinus edodes polysaccharide tablets
LPI: Lysophosphatidylinositol
LC–MS/MS: Liquid chromatography-tandem mass spectrometry
PI: Phosphatidylinositol
SYK: Spleen tyrosine kinase
p-SYK: Phosphorylated spleen tyrosine kinase
qPCR: Quantitative polymerase chain reaction;
RNA-seq: RNA sequencing
